# High-Tech Hip Implant for Wireless Temperature Measurements *In Vivo*

**DOI:** 10.1371/journal.pone.0043489

**Published:** 2012-08-22

**Authors:** Georg Bergmann, Friedmar Graichen, Jörn Dymke, Antonius Rohlmann, Georg N. Duda, Philipp Damm

**Affiliations:** Julius Wolff Institute, Charité – Universitätsmedizin Berlin, Berlin, Germany; University of Rochester, United States of America

## Abstract

When walking long distances, hip prostheses heat up due to friction. The influence of articulating materials and lubricating properties of synovia on the final temperatures, as well as any potential biological consequences, are unknown. Such knowledge is essential for optimizing implant materials, identifying patients who are possibly at risk of implant loosening, and proving the concepts of current joint simulators. An instrumented hip implant with telemetric data transfer was developed to measure the implant temperatures *in vivo*. A clinical study with 100 patients is planned to measure the implant temperatures for different combinations of head and cup materials during walking. This study will answer the question of whether patients with synovia with poor lubricating properties may be at risk for thermally induced bone necrosis and subsequent implant failure. The study will also deliver the different friction properties of various implant materials and prove the significance of wear simulator tests. A clinically successful titanium hip endoprosthesis was modified to house the electronics inside its hollow neck. The electronics are powered by an external induction coil fixed around the joint. A temperature sensor inside the implant triggers a timer circuit, which produces an inductive pulse train with temperature-dependent intervals. This signal is detected by a giant magnetoresistive sensor fixed near the external energy coil. The implant temperature is measured with an accuracy of 0.1°C in a range between 20°C and 58°C and at a sampling rate of 2–10 Hz. This rate could be considerably increased for measuring other data, such as implant strain or vibration. The employed technique of transmitting data from inside of a closed titanium implant by low frequency magnetic pulses eliminates the need to use an electrical feedthrough and an antenna outside of the implant. It enables the design of mechanically safe and simple instrumented implants.

## Introduction

### Friction of Implant Materials

High friction in joint implants and subsequent temperature rise during continuous activities, such as walking, may cause increased polyethylene wear, decreased polyethylene strength, or loosening of the cup in hip implants due to high frictional torque [1]. The natural cartilage has a coefficient of friction of 0.02 to 0.04 [2]. Articulating materials used for total joint replacement have higher friction. Coefficients reported in the literature are as follows: 0.04 to 0.05 for the combination Al_2_O_3_ - Al_2_O_3_, 0.05 to 0.055 for Al_2_O_3_ - UHMWPE, 0.06 to 0.07 for CoCrMo - UHMWPE, and 0.10 to 0.20 for CoCrMo - CoCrMo [3], [4], [5]. Moreover, a strong influence of the protein concentration in the synovia on friction was reported [5], especially for CoCrMo - CoCrMo.

### Synovia Properties

After joint replacement, a pseudo-synovial membrane is formed, which produces hyaluronic acid (HA), similar to the natural membrane. The synovia volume is small. In the hip joint, volumes of 2.7 ml in asymptomatic hips and 6.1 ml in fractured hips were reported [6].

The properties of synovia vary considerably, and synovia can lose its lubricating properties at high temperatures [7]. Synovia viscosity in natural joints depends on the type of joint disease [8]. Differences of at least a factor of 10 were determined between subjects and between healthy and osteoarthritic joints [9]. The most decisive factor for lubrication is the protein content in the synovia [10]. The lubrication ability of synovia from degenerative knee joints was worse than that of bovine serum [11], which may indicate that joint simulators do not actually mimic the real situation in hip or knee implants if a ‘standardized’ bovine serum is used [12], [13], especially if the temperature is kept constant at 37°C.

The cited literature indicates that individually varying synovia properties may strongly influence wear and temperature increases in replaced hip and knee joints during long-lasting activities, such as walking.

### Temperature in Hip and Knee Joints

*In vitro* temperature measurements on two intact human hip joints delivered a temperature increase of 2.5°C during simulated walking [14]. *In vivo* measurements of temperatures in the natural knee joint showed a 1°C increase in temperature after 20 minutes and 2°C after 40 minutes of walking [15]. Depending on the implant type and articulating materials, this increase was observed up to 7°C for a rotating hinge implant (CoCrMo - UHMWPE). In an analytical study, validated by simulator data, temperatures up to 51°C were found in CoCrMo - UHMWPE hip implants [16].

With instrumented implants, the forces and temperatures in Al_2_O_3_ - UHMWPE hip joints were measured in 5 subjects during 45 to 60 minutes of walking and bicycling [17]. After walking, the temperature rose up to 43.1°C in the patient with the lowest body weight. Another patient with a much higher body weight reached a joint temperature of only 40.0°C. In the only patient with bi-lateral implants, the temperature was 0.9°C lower with an Al_2_O_3_ cup than with a UHMWPE cup. After cycling, which caused 55% lower joint forces than walking, the temperatures were 1.5°C lower. We assume that the steady-state temperature after walking is closely correlated to the friction coefficient.

In a simulator, the surface temperature directly between a UHMWPE cup and an Al_2_O_3_ head was 45°C, but was 60°C with a CoCrMo head and 99°C with a zirconia ceramic [18]. These are temperatures at which synovia precipitates and loses its lubricating properties.

### Bone Necrosis

After heating rabbit thighs up to 42.5°C to 44.0°C using microwaves, strongly increased bone formation was observed [19]. After 4 minutes at 50°C, osteocytes were found to be irreversibly damaged [20]. Approximately 15–20% of the osteoblasts became necrotic after being exposed to 48°C for 10 minutes, while they withstood 45°C without damage [21]. After heating the superficial skull of rats to 48°C for 15 minutes, dead osteocyte areas were found, and the formation of new bone was delayed [22]. From the available literature on bone reactions to increases in temperature during drilling and sawing, it was concluded that 47°C is a critical temperature [23]. All of these studies investigated only the effect of non-recurrent high temperatures. Repeatedly acting heat may even cause cell damage at lower temperatures.

### Concepts of Instrumented Implants

Electronic components used for measurements with permanent implants, such as joint replacements, must be hermetically encapsulated. The optimal solution would be a complete enclosure by a metal [24] or ceramic [25] material. If an antenna or induction coil is placed outside the implant, only biocompatible materials are permitted [26], [27]. Certified pacemaker feedthroughs are then favorably used for connections to the implant electronics. Our force measuring hip implants employed an internal power coil and an external niobium antenna [28], [29], [30], [31]. Solutions using plastic-encapsulation for non-biocompatible electronic components [32], [33], [34] should only be used for non-permanent implants.

Power and signals could respectively be transferred to and from the implant by electro-magnetic fields. These fields are partly absorbed by a metal implant with the loss dependent on the alloy and frequency. Pure Ti, Al and V are paramagnetic with a relative magnetic permeability slightly greater than one. Implants made from such alloys only moderately weaken magnetic fields of low frequencies. However, ferromagnetic materials, such as Co or Ni, with a relative magnetic permeability of 80 to 600 almost completely shield the interior of an implant.

The loss caused by encapsulations made from Ti alloys is strongly frequency-dependent. A closed TiAl_6_V_4_ housing with a 2-mm wall thickness shields 23% of a magnetic field at 4 kHz but 53% at 10 kHz [30]. Any energy loss is accompanied by a temperature increase of the implant. Both the power consumption and the shielding loss must therefore be kept low. A titanium implant with an internal secondary power coil and transmitting antenna should use frequencies below 10 kHz for power as well as signal transfer. All transponder systems work at higher frequencies up to the GHz range and can therefore not be used inside a metallic implant. Locating transponder systems at the surface of a metallic implant [35] may cause problems for signal and energy transfer.

### Goals of this Work

The reported strong differences of friction coefficients, the individual variations of synovia properties, and the question of how well joint simulators mimic the *in vivo* loading conditions demonstrate the need to obtain *in vivo* information on the friction-induced temperature rise in joint implants.

The aim of the study was to design a temperature measuring hip implant with telemetric data transfer, which is completely safe for patients and can be used in a clinical study with a large number of patients. Furthermore, the technique described should be applicable for the instrumentation of other kinds of implants.

The following features were included: inductive power supply, inductive data transfer through the wall of the hermetically closed metallic implant, power consumption below 10 mW, measuring accuracy of 0.2°C, design based on a clinically well-proven implant type, and no requirement to change the surgical procedure.

## Methods

### Mechanical Design

**Figure 1:**
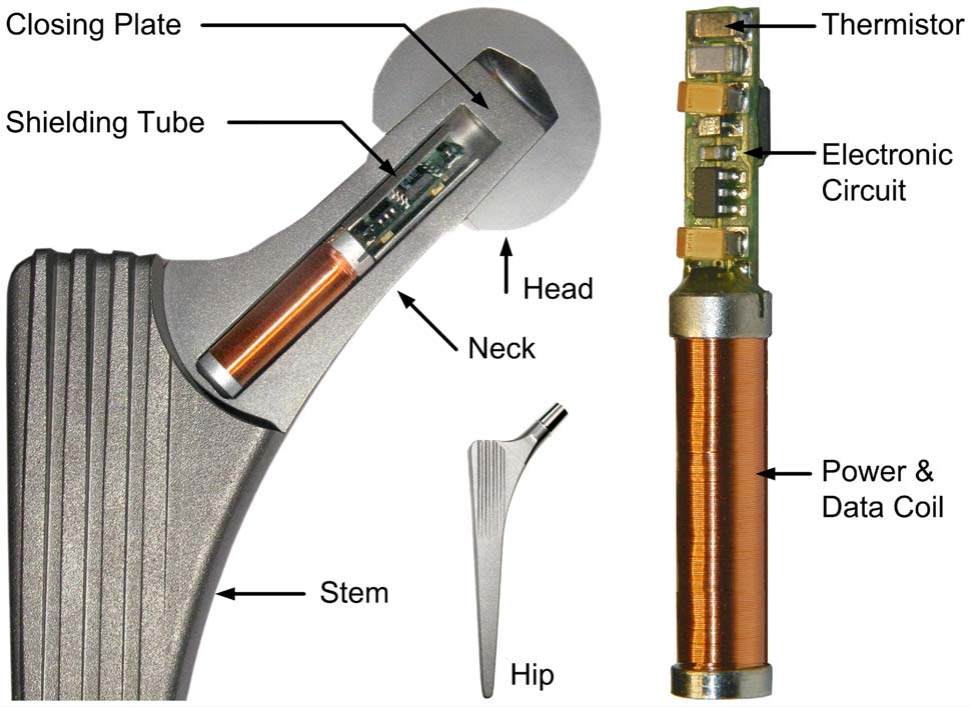
Cross-section of a model of the modified hip implant with a metal head. The temperature telemetry with thermistor, electronic circuit and power/data coil are placed inside the neck of the implant.

The non-cemented CTW™ Classic hip implant with a 12/14-mm cone (Merete Medical, Berlin, Germany) was used for instrumentation. It closely resembles implants of other manufacturers with very good clinical results [36]. The implant shape was only slightly modified between the neck and shaft to further increase its stability (Figure 1). A 6.2-mm-wide by 50-mm-long bore in the neck houses the temperature telemetry. At its top, a 5-mm-thick plate was welded using an electron beam (ENG Produktions-GmbH, Berlin, Germany) with a weld depth of 2.5 mm. The low required welding energy and the clamping of a massive copper block around the welding area facilitated temperature retention at the outside of the implant neck, at half of its length, below 80°C. The weld quality was checked for each produced batch by cutting samples, and the density of the welds was determined in a vacuum chamber. Fatigue strength of the implant stem and neck were tested according to [37], [38], [39] but with double the force levels in the neck test.

### Telemetry

**Figure 2:**
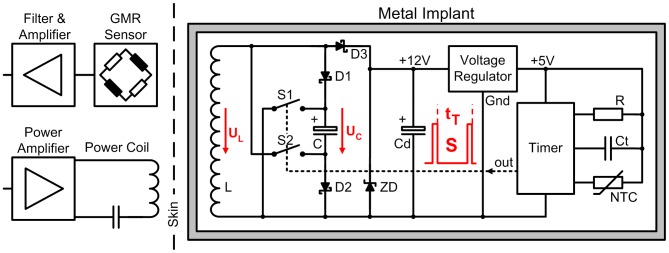
Principle of the temperature telemetry. Energy and temperature data are transferred through the titanium implant by induction.

The telemetry (Figure 2) is powered inductively at 4 kHz, as in our previous implants [29]. The internal coil L consists of 2700 loops on a core of PERMENORM 5000 H2 (µ_r_>12000, Vacuumschmelze). The induced voltage U_L_ is limited to 12.3 V by the Zener diode ZD, rectified by D3 and regulated to 5 V DC (Max 8881, Maxim).

A NTC thermistor (Epcos) serves as a temperature sensor. The ceramic capacitor C_t_ (Kemet) with COG/NPO parameters has a high Q, low K, a temperature-compensated dielectric, and stable electrical properties at varying voltage, temperature, frequency and time. Together NTC and C_t_ set a time constant t_T_, which triggers a timer (ICM 7242, Intersil). This timer produces the output signal S, a pulse train with temperature-dependent pulse intervals. The sampling rate is approximately 10 Hz at 60°C, 5 Hz at 37°C, and 2.1 Hz at 20°C.

**Figure 3:**
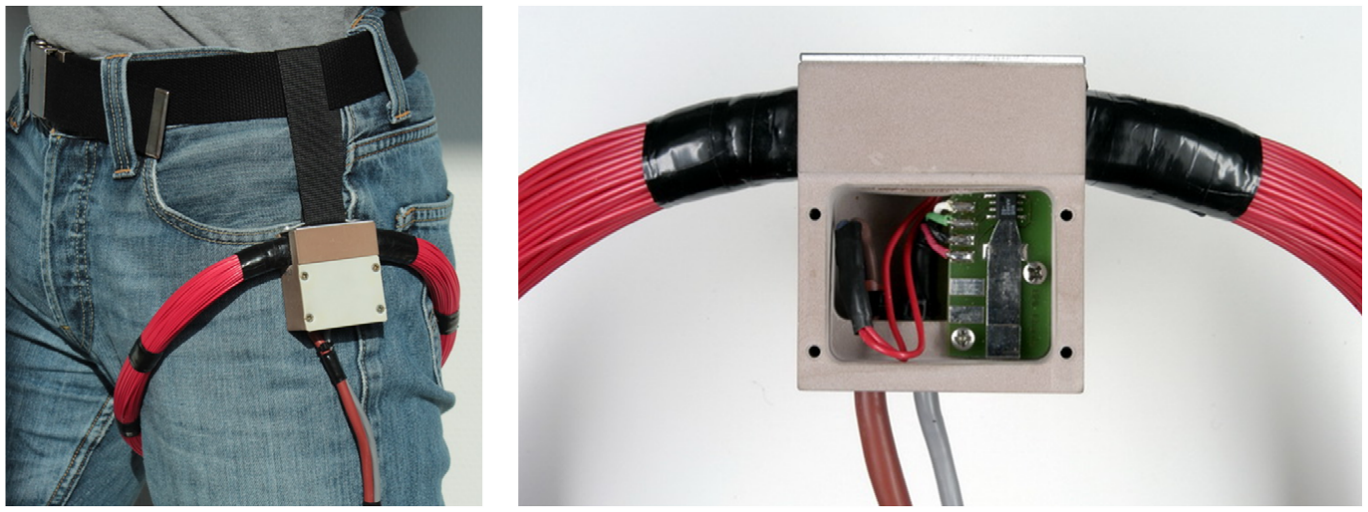
External equipment. The power coil with GMR sensor are fixed near the patient’s hip and connected to the external device TELETEMP.

**Figure 4:**
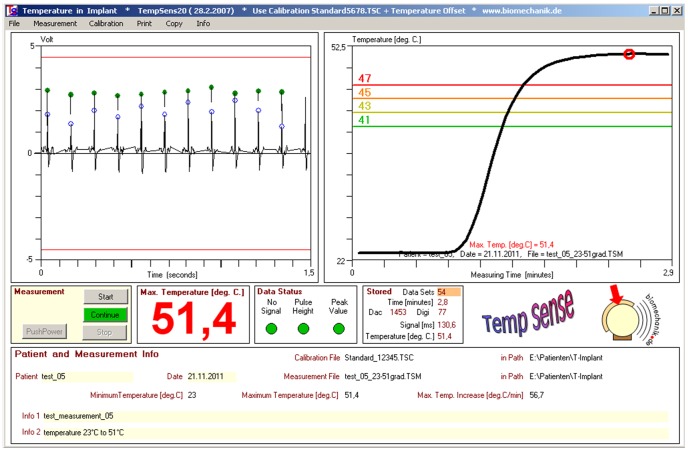
Measuring program. Screen shot from test measurements. Left: pulse signal from implant. Marked peak values and time points for counting temperature-dependent pulse intervals. Right: sudden temperature increase in a water bath.

Coil L is used not only for power transfer but also for signal transmission by superimposed magnetic pulses. The tantalum chip capacitor C is charged by L and the Schottky diodes D1, D2 to a maximum of U_C_ = 11.7 V DC. The electrical pulses of signal S close the digital FET switches S1 and S2 (FDC 6303N, Fairchild Semiconductor). C is then discharged over L resulting in magnetic pulses. After each pulse, both FET open and C is charged again. The transmitted pulses have a duration of 3 ms, and this telemetry circuit has a power consumption of 7 mW.

All active and passive components are surface mount devices on both sides of an 18×5 mm wide substrate (Figure 1). The NTC is positioned near the center of the prosthetic head. To shield the electronic components against the magnetic field, a tube of PERMENORM 5000 H2 with a wall thickness of 0.25 mm is slipped over the whole circuit and fixed with epoxy structural adhesive DP190 (3 M Scotch-Weld). The telemetry (40 mm long, 6.1 mm Ø) is fixed inside the prosthetic neck with DP190.

**Figure 5:**
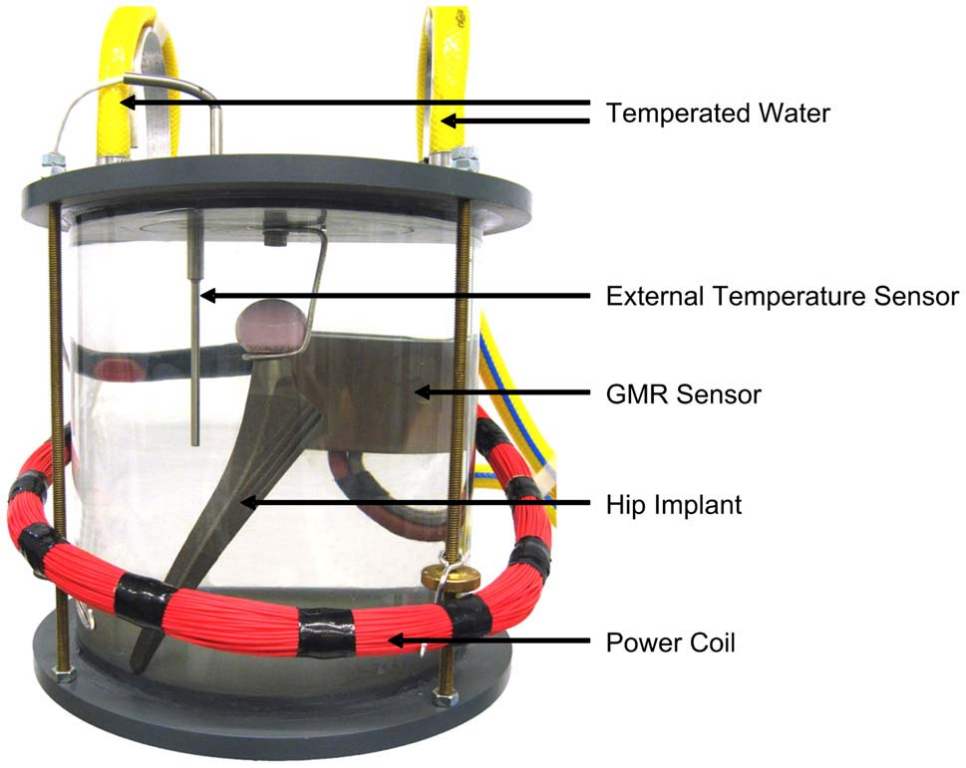
Implant calibration. The implants were calibrated in a circulating water bath at temperatures between 23°C and 58°C.

### External Measuring System

The specially developed unit ‘TELETEMP’ contains a power oscillator, amplifier and microcontroller (AVR-ATmega128, Atmel) with a USB interface and display. Because the transmitted magnetic pulses are low in power, a very sensitive sensor had to be chosen. This giant magnetoresistive (GMR) field sensor (AA002-02, NVE-Corporation) consists of a Wheatstone bridge and has an on-chip flux concentrator to increase its sensitivity along a specified axis.

Because the external powering field is much stronger than the field produced by the signal pulses, care must be taken that the GMR sensor measures as little as possible of the 4-kHz powering field. The spatial positions of GMR sensor and external induction coil (n = 210, D = 25 cm, L = 7.85 mH, C = 0.22 µF) are therefore fixed by a common, massive plastic housing (Figure 3). Within 5 cm from this housing the coil windings are furthermore inflexible. The sensor position is finally optimized by precisely adjusting the sensor board inside the housing.

The sensor signal is first high-pass-filtered (fc = 109 Hz, first-order) to remove the 50-Hz content. After a first amplification (AD8230, Analog Devices) a low-pass filter (1 kHz, 10^th^ order, LTC1569-6, Linear Technology Corp.) eliminates the remaining influence of the 4-kHz powering field. The gain of a second amplifier (AD8042, Analog Devices) can be set by a digital potentiometer (AD5282, Analog Devices). The pulses are converted to 12-bit digital values (MAX197, Maxim Integrated Products). The microcontroller checks the received signals S for missing pulses, amplifies their peak values to 3±1 V, counts their temperature-dependent time intervals t_T_ and sends S and t_T_ to a Windows PC.

### Power Supply

During the measurements the power coil is placed around the hip joint (Figure 3). The power oscillator generates a sinusoidal output voltage at 4±0.5 kHz. This frequency is permanently adapted to the resonance frequency of the coil. The oscillator output voltage and, thus, the magnetic field strength are controlled by the microcontroller. Primary and secondary power coils are fixed to the thigh and the femur, respectively. Except from soft tissue deformations they therefore move in the same way during walking and the induced supply voltage varies by not more than 5%. Using an induction frequency of 4 kHz with loose air-coupled coils a shift of the titanium implant is not detectable by the primary power coil.

The voltage U_C_ and, thus, the strength of the transmitted signal pulse directly depend on the magnetic field strength. For U_C_ below 5 V, the circuit is unstable, and the pulses cease. Levels above 11.7 V are prevented using Zener diodes. The range of the internal voltage U_C_ is controlled by analyzing the amplitude of the received pulses. Beginning with a high amplification, the magnetic field strength is reduced until the pulse height begins to decrease; U_C_ is then at its upper limit of 11.7 V. When the pulses vanish, U_C_ has reached its lower limit of 5 V. Based on these two values, U_C_ is set to 7.5 V. The operating range of 6 V to 9 V is a compromise between sufficient signal strength and the minimal power dissipation. This range allows position changes between the signal source inside the implant and the power coil around the leg without endangering the power supply. If U_C_ nevertheless exceeds one of its borders, U_C_ is automatically re-adjusted.

### Data Processing

The evaluation program is written in Visual Basic (Figure 4). Signal S is displayed, and its peak values are marked as well as the times used for counting t_T_. The temperature is calculated from previously obtained calibration data and charted for visual control.

### Calibration

**Figure 6:**
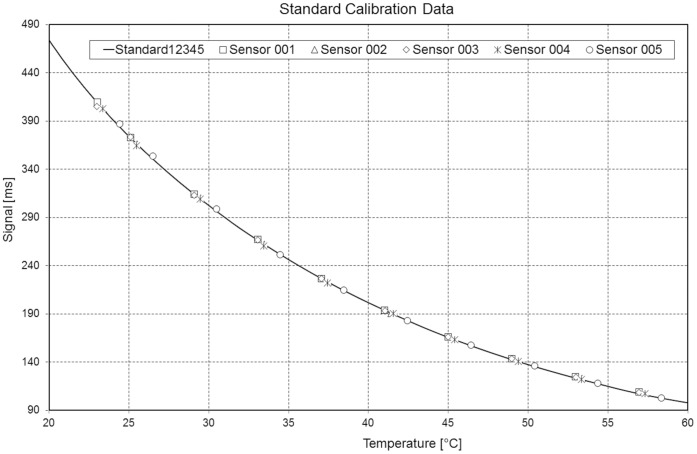
Standard temperature curve. The temperature-dependent signals of 5 implants are plotted.

For calibration, 5 prostheses were placed in a circulating water bath at 10 different temperatures between 23°C and 58°C (Figure 5). The temperature was adjusted with an accuracy of 0.1°C and measured close to the implant with an accuracy of 0.01°C (9540, Guildline Instruments). From this data, an average polynomial temperature curve, Temperature = f(t_T_), was calculated (Figure 6). Only an offset temperature at 37.5°C must later be determined for the implants used in patients.

## Results

### Implant Safety

Only prostheses with neck lengths XS (extra-small), S (small), M (medium) and L (large) will be implanted. For fatigue tests, a longer neck length of XL (extra-large) was used. Under this more severe condition, the implant stem passed the fatigue tests [37]. Then, the neck was tested with a maximum load of 5.3 kN during 10 million cycles [38]. This load was further increased every 1 million cycles in steps of 1 kN, without failure, up to 13 kN. This load was more than 2 times higher than the standard force for testing implant necks. The mechanical tests were performed by EndoLab GmbH (Rosenheim, Germany), and the sterilization process was certified by Vanguard AG (Berlin, Germany).

The telemetry system described was approved by BerlinCERT (Berlin, Germany) using standards of both EU Directive 90/385/EWG (AIMDD, Active Implantable Medical Device Directive) and EU Directive 93/42/EWG (MDD, Medical Device Directive).

A planned clinical study with 100 patients will begin after receiving approval from our Ethics Committee.

### Measuring Accuracy

The measuring accuracies were determined at 37.5°C, 43°C, and 50°C. The measured temperatures were compared with readings from a thermometer with an accuracy of 0.01°C. The error of the calibrated implants was always below 0.1°C.

To determine how fast the instrumented implants are able to detect temperature changes, a prototype was placed in cold circulating water. Then, boiling water was quickly added such that the temperature rose from 23°C to approximately 51°C. This temperature increase was recorded by the implant within 70 s (Figure 4).

## Discussion

The clinical study on temperature rise in hip implants is planned with 100 patients. The shape of the original implant was unchanged by the instrumentation, and thus, current surgical procedures can be used. Therefore, the same good clinical success, such as with other Spotorno-like implants, can be expected.

Investigations will be performed with 4 different combinations of head and cup materials and 2 different head sizes. This data will allow us to answer the following questions: a) Are certain material combinations producing such high temperatures that implant fixation of patients with badly lubricating synovia is endangered? b) How much do the lubrication properties of synovia differ individually? c) Can patients at risk be identified by intra-operative synovia tests? d) Do joint simulators deliver realistic results for friction and wear?

Our previous instrumented joint implants (www.OrthoLoad.com), with multi-channel telemetries for load measurements, required higher sampling rates and a signal transmission by radiofrequency. Because such signals are shielded by the metallic implant, electrical feedthroughs and an antenna outside of the implant were needed, which had to be biocompatible and protected mechanically. The low frequency magnetic pulses, now used for data transfer, are only marginally weakened by titanium implants and can therefore be transmitted through the metallic wall. This enables the design of mechanically safe and simple instrumented orthopedic implants. In addition, the described technique is also well suited for measuring strains or detecting implant loosening by frequency analyses.

The low power consumption of 7 mW prevents a temperature increase by the inductive power supply. The transmission rate of 5 Hz is sufficiently high for measuring temperatures, and the measuring error of 0.1°C is lower than expected.

The transmission rate can be increased, for example, up to 50 Hz, with good accuracy. For measuring fast changing signals this rate may still be too low. Sampling can be accelerated, however, if the signals are not transmitted in real-time. Instead, they can be measured at a high rate, stored temporarily in the memory of a microcontroller and transmitted at a lower rate directly after the measurement. Transmitting signals at a rate that is ten times lower would allow sampling of 1 signal of at least 500 Hz or of several signals at a lower rate.

As described, the GMR sensor has to be adjusted carefully, and its signal is filtered not to measure the 4-kHz powering field but to measure only the magnetic pulses produced by the implant. Currently the signal-receiving circuit is changed such that the signal will only be received at time points when the powering field is close to the zero crossing, which will ensure that the quality of the signals is significantly less dependent on the exact adjustment of the power coil and the GMR sensor.
